# Genetic Modifiers of White Blood Cell Count, Albuminuria and Glomerular Filtration Rate in Children with Sickle Cell Anemia

**DOI:** 10.1371/journal.pone.0164364

**Published:** 2016-10-06

**Authors:** Beverly A. Schaefer, Jonathan M. Flanagan, Ofelia A. Alvarez, Stephen C. Nelson, Banu Aygun, Kerri A. Nottage, Alex George, Carla W. Roberts, Connie M. Piccone, Thad A. Howard, Barry R. Davis, Russell E. Ware

**Affiliations:** 1 Cincinnati Children’s Hospital Medical Center, Cincinnati, OH, United States of America; 2 Baylor College of Medicine, Houston, TX, United States of America; 3 University of Miami, Miami, FL, United States of America; 4 Children’s Hospitals and Clinics of Minnesota, Minneapolis, MN, United States of America; 5 Cohen Children’s Medical Center, New Hyde Park, NY, United States of America; 6 St. Jude Children’s Research Hospital, Memphis, TN, United States of America; 7 University of South Carolina, Columbia, SC, United States of America; 8 Case Western Reserve University, Cleveland, OH, United States of America; 9 University of Texas School of Public Health, Houston, TX, United States of America; Université Claude Bernard Lyon 1, FRANCE

## Abstract

Discovery and validation of genetic variants that influence disease severity in children with sickle cell anemia (SCA) could lead to early identification of high-risk patients, better screening strategies, and intervention with targeted and preventive therapy. We hypothesized that newly identified genetic risk factors for the general African American population could also impact laboratory biomarkers known to contribute to the clinical disease expression of SCA, including variants influencing the white blood cell count and the development of albuminuria and abnormal glomerular filtration rate. We first investigated candidate genetic polymorphisms in well-characterized SCA pediatric cohorts from three prospective NHLBI-supported clinical trials: HUSTLE, SWiTCH, and TWiTCH. We also performed whole exome sequencing to identify novel genetic variants, using both a discovery and a validation cohort. Among candidate genes, *DARC* rs2814778 polymorphism regulating Duffy antigen expression had a clear influence with significantly increased WBC and neutrophil counts, but did not affect the maximum tolerated dose of hydroxyurea therapy. The *APOL1* G1 polymorphism, an identified risk factor for non-diabetic renal disease, was associated with albuminuria. Whole exome sequencing discovered several novel variants that maintained significance in the validation cohorts, including *ZFHX4* polymorphisms affecting both the leukocyte and neutrophil counts, as well as *AGGF1*, *CYP4B1*, *CUBN*, *TOR2A*, *PKD1L2*, and *CD163* variants affecting the glomerular filtration rate. The identification of robust, reliable, and reproducible genetic markers for disease severity in SCA remains elusive, but new genetic variants provide avenues for further validation and investigation.

## Introduction

Despite sharing the same deleterious genetic mutation in the *HBB* beta globin gene, persons with homozygous HbSS (sickle cell anemia, SCA) have marked variability in their laboratory profiles and clinical disease expression.[1] Understanding the phenotypic variability of SCA is a desirable goal, since the identification of children with increased likelihood of severe disease manifestations could prompt early intervention with targeted and preventive therapy. Further, the elucidation of biochemical and genetic pathways contributing to the observed inter-patient variation might allow novel therapeutic interventions. To date, however, this goal has been elusive and relatively few validated biomarkers of disease expression have been accepted for patients with SCA.

Landmark studies from the United States Cooperative Study of Sickle Cell Disease (CSSCD) documented that simple laboratory measurements including fetal hemoglobin (%HbF), total white blood cell (WBC) count, and steady-state hemoglobin concentration (Hb) have predictive value for clinical complications and even mortality.[2–6] Subsequent studies have identified additional biomarkers of disease severity in SCA, such as microalbuminuria,[7] elevated transcranial Doppler (TCD) velocities,[8] and tricuspid regurgitant jet velocity;[9] these measures of organ function reflect acute and chronic damage, and confer risks of both morbidity and mortality.[8–10]

Genetic variants influencing laboratory and clinical markers of disease severity in SCA could be useful to help risk-stratify patients, enhance early screening efforts, and provide targeted therapeutic interventions for patients before the onset of irreversible organ damage. Similar to laboratory biomarkers, robust and reproducible genetic risk factors associated with SCA disease complications have been difficult to identify. With the notable exceptions of alpha-thalassemia trait,[11] UGT1A1 promoter variants,[12] and HbF-modifying *BCL11A* variants,[13] most other purported genetic markers affecting SCA disease severity have been challenging to replicate and validate, and none is routinely used in clinical practice.

With the advent of new genetic risk factors identified and validated for the general population, especially among African Americans, we hypothesized that several of these genetic variants could also modify the laboratory and clinical phenotype of SCA. Specifically, we hypothesized that genetic polymorphisms reported to affect the total WBC count or influence the development of early renal disease would be reproducible in children with SCA. The WBC and the related absolute neutrophil count (ANC) were selected as important biomarkers because of their documented importance in overall clinical severity and mortality of SCA,[2–4] and their recognized influence on the hydroxyurea maximal tolerated dose (MTD).[14,15] Genetic variants known to affect the WBC count, including polymorphisms in *DARC*, *PSMD3-CSF*, *CXCL2*, and *CDK6* genes[16–18] have not been carefully investigated in children with SCA. Similarly, the presence of albuminuria and elevated glomerular filtration rate (GFR) were selected as important biomarkers of early renal disease, since they portend further decline in renal function and eventual end-stage renal disease.[10,19] Known genetic variants in *APOL1*, *eNOS* and *CUBN* could influence the onset of renal dysfunction in SCA and contribute to sickle cell nephropathy.[20–22]

We now provide results of both a targeted candidate gene approach and an additional large unbiased whole exome analysis of genetic variants influencing the WBC and ANC counts, plus albuminuria and GFR, in three large prospective cohorts of children with SCA. Using both a discovery and a validation cohort for each genotype-phenotype association, our results document several common genetic variants that influence these important SCA phenotypes, and thus may affect overall clinical disease expression and prognosis. In addition, our analyses identify several novel genetic variants that warrant further validation to help elucidate disease pathophysiology and aid future therapy of SCA complications.

## Methods

### Study populations

Three prospective NHLBI-funded research studies involving children with SCA were used for patient samples and clinical data: (1) Hydroxyurea Study of Long-term Effects (HUSTLE, NCT00305175), which enrolled children with recurrent vaso-occlusive events and investigated hydroxyurea pharmacokinetics as well as long-term clinical, cellular and molecular effects of hydroxyurea exposure at MTD, with an average enrollment age of 10 years (N = 181);[15] (2) Stroke With Transfusions Changing to Hydroxyurea (SWiTCH, NCT00122980), a Phase III, multicenter trial comparing alternative therapy (hydroxyurea and phlebotomy) to standard treatment (transfusions and chelation) for secondary stroke prevention and iron management, respectively, in children with prior stroke and iron overload, with an average enrollment age of 13 years (N = 137);[23] and (3) TCD With Transfusions Changing to Hydroxyurea (TWiTCH, NCT01425307), a Phase III multicenter, non-inferiority trial comparing alternative therapy (hydroxyurea) to standard treatment (transfusions) for maintenance of TCD velocities in children with abnormal TCD velocities, with an average enrollment age of 9 years, (N = 131).[24] Written informed consent for genetic analyses was obtained from parents or legal guardians of minors for each clinical trial, along with assent from minors as required by each local IRB. All genomic studies were then reviewed and IRB-approved by an additional CCHMC research protocol.

### Phenotypes

Blood and urine samples were collected from children with SCA at the time of enrollment, per protocol, and always before initiation of hydroxyurea therapy. All laboratory values were de-identified and recorded in centralized databases. Fewer than 10% of children enrolled in HUSTLE had recent transfusion exposure, while all SWiTCH and TWiTCH participants were receiving chronic transfusions at the time of enrollment. Hydroxyurea MTD was determined as previously described, representing a stable daily dose that causes adequate marrow suppression without hematological or other toxicity.[25]

For the measurement of WBC and ANC, all complete blood counts were performed locally with standard automated leukocyte differentials. For the renal phenotypes, albuminuria was analyzed locally for HUSTLE and centrally for TWiTCH, but not available for SWiTCH participants. Albuminuria was measured quantitatively and reported as mg albumin per gram of creatinine with normal <30 mg/g, microalbuminuria 30–300 mg/g, and macroalbuminuria >300 mg/g creatinine. Standard urinalysis and serum creatinine were performed centrally on all participants, and measures of cystatin C were performed using nephelometry as described.[26] In HUSTLE, GFR was measured directly by ^99m^Tc-labeled diethylenetriaminepentaacetic acid (DTPA) plasma clearance as described[27] while in TWiTCH an estimated GFR (eGFR) was calculated using a published equation based on serum creatinine.[28]

### Genotypes

Genomic DNA was extracted from whole blood using standard techniques, and stored at -20°C until analysis; all samples were de-identified and specific genotypes could be linked to phenotypes only through the main study databases. Candidate genes were identified from the published literature based on influence on the phenotypes of interest, to identify single nucleotide polymorphisms (SNPs) and other variants[16,17,20,21,29–31] used to genotype patient samples (S1 Table). In most cases, genotyping was performed using TaqMan-based polymerase chain reaction (PCR) with commercially available or customized primers (Applied Biosystems, Foster City CA). DNA samples were tested with ~10% duplicates as quality control; in each assay, >95% samples were required to give a genotype call to allow the results to be included. To genotype the *APOL1* 6-bp deletion, PCR amplification was followed by bidirectional Sanger-based sequencing of the product. For the *eNOS* 27-bp nucleotide variable repeat, the PCR product was resolved using polyacrylamide gel electrophoresis and scored based on fragment size.

### Whole exome sequencing (WES)

For the WBC phenotype, all available samples with WES data (n = 383) were randomly divided into an initial discovery cohort (n = 256) and a subsequent validation group (n = 127). To identify potentially novel genes associated with early renal disease, we analyzed DNA from patients who had both measurements of GFR and available WES data (n = 187); an initial discovery cohort included patients with measured DTPA GFR (n = 62) and the validation cohort included patients with calculated eGFR (n = 125). The primary strategy was to find variants that maintained association between the discovery and validation analyses for each phenotype. Linear regression analysis was used to independently test the association of each common (minor allele frequency >2%) non-synonymous variant in both the discovery and validation cohorts.

Whole exome sequence analysis was performed as previously described.[32,33] In brief, WES data were generated using NimbleGen VCRome 2.1 capture reagents followed by sequencing on an Illumina platform. Individual sequence reads were aligned to a reference human genome and variants were annotated. All 383 samples within this study passed stringent WES quality control parameters with an average of 92% of the targeted exonic regions sequenced at >20x coverage per individual. A project level variant call format (VCF) was generated for all samples, and included variants that were present in at least one sample. Variants with more than 5% missed genotyping calls were excluded from analysis.

### Statistical analysis

The average age and ANC levels were elevated in all three cohorts, reflecting the generalized inflammation observed in SCA and no normalization from transfusion therapy. However, the ANC was slightly but significantly higher in patients enrolled in SWiTCH compared to the other two studies; accordingly, for the WBC and ANC phenotypes, the regression analysis included adjustment for age, sex, and study cohort. A Box-Cox transformation was used to attain a normal distribution of the WBC, ANC, DTPA GFR, and eGFR phenotypes. Linear regression analysis was used to test associations with each transformed phenotype, including fixed effects for all variant genotypes and covariate correction for age, sex, enrolled study, and population stratification. Population stratification was assessed by principal component analysis using Eigenstrat. An additive genetic model was used for all genetic testing. For the WES data analysis, we performed quality control filtering steps including: SNP missingness check; removal of sex chromosomes; monomorphic site, synonymous and intronic variant removal; excess heterozygosity filter; and 2% minor allele frequency cut-off. No adjustments were made for multiple testing.

## Results

### Leukocyte phenotype

All study participants had baseline WBC and ANC values available for analysis. For the entire cohort (n = 429), the average age (mean ± SD) was 10.7 ± 4.1 years, and leukocytosis was observed with average WBC = 13.8 ± 4.4 x 10^9^/L and ANC = 7.6 ± 3.2 x 10^9^/L.

Candidate SNP analysis results are summarized in Table 1. The *DARC* SNP rs2814778 regulating Duffy antigen expression had a minor allele frequency of 15.1%, similar to published reports in the general African-American population.[16,34] A total of 111 children were Duffy-positive (Fy+), either homozygous or heterozygous for the T allele (Fya+b+, Fya+b-). Duffy-positive children had significantly higher WBC and ANC, compared to Duffy-negative children (Fya-b-), 14.6 versus 13.5 x 10^9^/L and 8.5 versus 7.2 x 10^9^/L (p = .023 and p = .008, respectively).

**Table 1:** Association of candidate genes with WBC and ANC.

Gene	dbSNP ID	MAF	Reference	WBC		ANC	
				Beta value (SD)	p value	Beta value (SD)	p value
*DARC*	rs2814778	.151	.205	1.02 (0.43)	.023	0.92 (0.31)	.008
*DARC*	rs12075	.059	.148	0.75 (0.63)	.259	0.97 (0.45)	.039
*IFI16*	rs4657616	.069	.098	1.22 (0.62)	.061	0.62 (0.44)	.120
*PSMD3-CSF*	rs4065321	.386	.352	0.39 (0.32)	.182	0.32 (0.23)	.146
*CDK6*	rs445	.188	.115	-0.21 (0.41)	.597	-0.15 (0.28)	.725
*CXCL2*	rs9131	.202	.295	-0.27 (0.41)	.436	-0.14 (0.23)	.689

The reference frequencies were taken from 1000 genomes in African American individuals (n = 61). MAF, minor allele frequency; SD, standard deviation. The beta value indicates the average change in values per allele of each variant.

Two other candidate SNPs, rs12075 and rs4657616, are known to be in moderate linkage with the DARC rs2814778 SNP (rs12075 D’ = 0.99, r^2^ = 0.36; rs4657616 D’ = 0.91, r^2^ = 0.34), and in our analyses both the rs12075 G and rs4657616 G polymorphisms were always found in combination with the rs2814778 T polymorphism (Duffy positive state). The SNP rs12075 G polymorphism, located in the *DARC* gene and conferring Duffy blood group antigen Fy^a^ expression, was associated with higher ANC than Duffy antigen Fy^b^ expression (8.8 versus 7.4 x 10^9^/L, p = .039). However, since every child carrying the rs12075 minor allele was also Duffy positive, this association could be mostly driven by the rs2814778 SNP. After further regression analysis with covariate correction for rs2814778 genotype, there was no longer any association of ANC or WBC with rs12075. In contrast, the rs4657616 G polymorphism, located in proximity to *DARC*, was only nominally associated with WBC and ANC (Table 1). None of the other candidate SNPs was found to have significant influence on the baseline WBC or ANC values.

These genetic variants affecting WBC and ANC were next investigated for an association with the hydroxyurea MTD. Due to the randomized trial design of SWiTCH and TWiTCH, not all study participants received hydroxyurea; MTD data were available on a total of 224 children who averaged 25.6 ± 4.5 mg/kg/day.[15,23,24] The hydroxyurea MTD was associated with higher pre-treatment WBC and ANC levels (p = 0.007 and 0.003, respectively), but not with any of the candidate genetic polymorphisms.

An unbiased genetic association study using whole exome data was then performed. After variant filtering, a total of 33,324 non-synonymous exonic variants with minor allele frequency >2% were identified. The quantile-quantile (Q-Q) plots of the p values from the WBC and ANC association tests showed good agreement with their expected distributions, indicating there were few biases due to population stratification, cryptic relatedness, study enrollment, or patient age. No single genetic variant for either phenotype passed exome-wide significance (p<5 x 10^−8^). However, to identify novel coding genetic variants with potential association with WBC and ANC levels, we selected variants with at least moderate association (p < .005) in the discovery cohort and performed replication testing of these variants in the validation cohort (p < .05).

For the WBC phenotype there were 133 variants with significant association in the discovery group; only 6 of these variants maintained association in the validation cohort (Table 2). Similarly, for the ANC phenotype there were 141 associated variants identified in the discovery cohort; again only 6 of these variants had replicated association in the validation cohort (Table 2). For both WBC and ANC phenotypes, a meta-analysis was then performed for each of the 11 variants consistently associated with either phenotype in both the discovery and validation sample groups, to provide overall p-values and estimated effect sizes (Table 2). The only variant that was significantly associated with both ANC and WBC after our discovery and validation analyses was rs28376707 in the *ZFHX4* gene, (overall p-values < .0005 for both WBC and ANC). This variant confers an A1639V amino acid change and may represent a novel polymorphism that influences both WBC and ANC. In this WES analysis, the *DARC* rs2814778 SNP was only significantly associated with WBC and ANC when there was no covariate correction for population stratification, and only in the overall meta-analysis (p = .025 and p = .008, respectively).

**Table 2:** Genetic variants identified by WES associated with either WBC or ANC.

#	Gene	Chr	Position	dbSNP ID	MAF	WBC Overall p-value	WBC Effect size (SE)	ANC Overall p-value	ANC Effect size (SE)
***1***	***RRAD***	16	66958886	rs7198458	.131	.000033	-0.216 (0.051)	.00157	-0.165 (0.051)
***2***	***AK9***	6	109885475	rs10499052	.061	.000088	0.201 (0.051)	.00019	0.191 (0.051)
***3***	***LAMA2***	6	129824406	rs73599293	.066	.00011	0.198 (0.051)	.00578	0.142 (0.051)
***4***	***EXD3***	9	140277826	rs13291830	.236	.00023	-0.189 (0.051)	.00118	-0.167 (0.051)
***5***	***ITGA11***	15	68609647	rs2271725	.313	.00034	0.183 (0.051)	.02788	0.112 (0.051)
***6***	***ZFHX4***	8	7764073	rs28376707	.085	.00045	0.179 (0.051)	.000082	0.202 (0.051)
***7***	***NES***	1	156641537	rs951781	.094	.000011	0.225 (0.051)	.0000003	0.263 (0.051)
***8***	***FAM221A***	7	23728931	rs35495590	.039	.00160	-0.161 (0.051)	.000027	-0.216 (0.051)
***9***	***MAN2B1***	19	12767849	rs34544747	.238	.00515	0.143 (0.051)	.000082	0.202 (0.051)
***10***	***OR10G9***	11	123894119	rs12366219	.112	.00042	-0.181 (0.051)	.00024	-0.189 (0.051)
***11***	***CERS4***	19	8326866	rs17160348	.146	.00203	0.158 (0.051)	.00025	0.188 (0.051)

The gene name and dbSNP ID are given for each polymorphism, along with the MAF in the whole cohort (n = 383). From the discovery and validation analysis, we identified six variants associated with WBC that passed the discovery and validation thresholds (#1–6). Similarly, we identified six variants associated with ANC after discovery and validation (#6–11). The *ZFHX4* variant was significantly associated with both WBC and ANC.

### Renal phenotype

The phenotype of albuminuria was available for 79 unrelated children enrolled in HUSTLE and 118 in TWiTCH, for a total of 197 participants (98 males and 99 females) with an average age of 10.0 ± 3.5 years. Microalbuminuria was present in 13.2% of patients overall, including 16.5% in HUSTLE and 11.0% in TWiTCH. The average age of patients with microalbuminuria was 11.0 years of age and there was a weak association of age with risk of albuminuria (p = .07). The only hematological parameter associated with albuminuria was lower WBC and ANC levels (p = .039 and p = .024, respectively). Among the HUSTLE participants with available measured GFR (n = 82), the average value was 155.9 ± 37.5 mL/min/1.73m^2^ (median 158.0, range 77.0–308.0 mL/min/1.73m^2^), which was significantly correlated with the eGFR in these same patients (r = .32, p = .003). HUSTLE patients with microalbuminuria (n = 13) had higher measured GFR compared to those without microalbuminuria (173.9 ± 50.3 versus 152.7 ± 34.1 mL/min/1.73m^2^; p = .07). Among TWiTCH participants with available calculated GFR (n = 138), the estimated value was 160.2 ± 67.7 mL/min/1.73m^2^ (median 160.2, range 77.5–496.6 mL/min/1.73m^2^). The candidate SNP analysis is summarized in Table 3. *APOL1* genetic variants were common (G1 allele frequency = 21.9%, G2 allele = 16.0%) and similar to published frequencies in adults.[22,31,35] Children with at least one *APOL1* G1 allele had an increased risk of albuminuria (Table 3, p = .017). Homozygotes for the G1 allele also had an increased risk of albuminuria (15.3% vs. 4.4% of participants; p = .033). There was nominal association of at least one *APOL1* G2 allele with protection from albuminuria (p = .048) in our cohort.[31] Analysis for any additive effect of the *APOL1* variants was negative; G1/G2 compound heterozygous individuals had no significant increased risk of microalbuminuria. Conversely, the presence of *DARC* rs2814778 that confers the Duffy antigen positive (Fy+) phenotype was associated with a small protective effect against albuminuria (Table 3, p = .046). None of the other polymorphisms analyzed achieved statistical significance. There was no association of any candidate polymorphisms with serum creatinine, blood pressure or serum cystatin C levels (data not shown).

**Table 3:** Association of candidate gene variants with the presence of albuminuria.

Gene	dbSNP ID	MAF	Albuminuria (Additive) p value	Variant frequency		Odds Ratio (C.I)
				Cases	Controls	
*APOL1* G1	rs73885319	.219	.017	32.0%	20.3%	2.51 (1.18–5.46)
*APOL1* G2	rs71785313	.160	.048	10.0%	16.0%	0.36 (0.12–0.99)
*DARC*	rs2814778	.136	.046	4.2%	15.7%	0.29 (0.07–0.99)
*eNOS* 4a	VNTR	.312	.333*	31.5%	22.7%	-
*eNOS*	rs1799983	.125	.883	14.6%	12.2%	0.93 (0.36–2.43)
*eNOS*	rs2070744	.156	.263	10.4%	16.3%	0.58 (0.21–1.59)
*CUBN*	rs7918972	.162	.345	12.5%	16.8%	0.64 (0.24–1.71)
*CUBN*	rs1801239	.027	.199	0.0%	3.1%	-

A case:control association test was performed for each polymorphism using a correlation trend test with correction for age and sex.

*4a variant compared to all other *eNOS* variants.

Whole exome sequence data was available for 22 patients with albuminuria and 145 patients without albuminuria; we observed that one additional non-synonymous variant in *APOL1* (rs60910145) was associated with increased risk of albuminuria (p = .038); this variant is in strong linkage with the rs73885319 variant (D’ = .97, r^2^ = .94) and in combination defines the *APOL1* G1 risk allele. There were no other *APOL1*, *MYH9*, *CUBN* or *eNOS* WES variants with even minimal association with albuminuria (p < .05).

To identify potentially novel genes associated with early renal disease, we opted to investigate GFR due to the low prevalence of albuminuria in our patient cohort. All available WES data for patients with varying degrees of GFR (n = 187) were analyzed using both a discovery and a validation cohort. In the discovery cohort (n = 62), we identified 1603 non-synonymous gene variants associated with DTPA GFR (p < .05).

Using independent patients with eGFR values as the validation cohort (n = 125), 36 variants in 34 genes maintained their association (p < .05). Of the genes with strongest association, variants in the *PKD1L2*, *AGGF1*, *CYP4B1*, *CUBN*, and *TOR2A* genes were of particular interest due to their proposed functions (Table 4). We also identified a variant in the *CD163* gene that approached significance in our validation cohort (p = .059) with significant association in the overall cohort.

**Table 4:** Coding variants associated with glomerular filtration rate.

Gene	Chr	Position	dbSNP ID	MAF	DTPA GFR		eGFR		Meta-Analysis	
					p-value	Beta-Value (SD)	p-value	Beta-Value (SD)	p-value	Effect size (SE)
***PKD1L2***	16	81181120	rs76056952	.031	.0443	-24.4 (13.3)	.0009	-61.8 (28.9)	.00011	-0.28 (0.07)
***AGGF1***	5	76332468	rs72765108	.083	.0014	35.7 (10.5)	.0127	44.8 (17.1)	.00011	0.28 (0.07)
***TOR2A***	9	130494486	rs114990094	.031	.0475	-32.5 (17.4)	.0031	-63.9 (26.8)	.00037	-0.26 (0.07)
***CUBN***	10	16893332	rs111265129	.039	.0469	-48.8 (25.8)	.0160	-42.4 (20.1)	.00186	-0.23 (0.07)
***CYP4B1***	1	47280852	rs12094024	.073	.0223	37.6 (13.3)	.0369	42.7 (17.1)	.00256	0.22 (0.07)
***CD163***	12	7640395	rs61729510	.042	.0091	68.2 (20.3)	.0588	51.8 (19.0)	.00237	0.23 (0.07)

From the discovery (DTPA GFR) and validation (eGFR) analysis, five variants were identified that passed both discovery and validation significance thresholds (p < .05). An additional variant in CD163 closely approached significance in the validation cohort. Beta-Value refers to the average change in the GFR, per allele of each listed variant.

## Discussion

In the new era of “personalized medicine” the prospective investigation of genetic differences that influence laboratory and clinical phenotypes, variability in medication dosing, and treatment responses, is warranted. Genetic modifiers could be used as predictors of disease severity, providing the means to risk-stratify patients, enhance early screening efforts, and provide targeted and optimal therapeutic interventions. In SCA, the observed phenotypic variability is likely influenced by a combination of genetic modifiers, although many reported SNPs and other variants have not yet been validated or shown to have clinical utility.

Simple laboratory phenotypes with clear predictive importance for clinical morbidity in SCA were investigated. Because the sample sizes were not large, only a limited number of candidate gene variants were investigated, and the unbiased WES analysis was divided randomly to include both a discovery and validation cohort for each phenotype. Although the association p-values did not meet exome-wide significance thresholds, the patient cohorts had accurate quantitative phenotypes, and several genetic associations linked to leukocytosis and renal disease were validated.

The leukocytosis observed in SCA is thought to reflect generalized inflammation, due to repeated and chronic endothelial damage and subsequent ischemia-reperfusion injury.[34] WBC levels are also associated with higher hydroxyurea MTD,[14] presumably by avoiding dose-limiting toxicities that prevent dose escalation.[36] Many individuals of African descent have ANC levels <1.5 x10^9^/L due to a strong genetic influence from two *DARC* polymorphisms.[18] For the first time, these genetic modifiers are shown to also affect children with SCA; specifically Duffy antigen positive (Fy+) children had a modest but significantly higher WBC and ANC compared to Duffy-negative children (Fy-). A small but significant association between Fy^a^ and Fy^b^ expression with ANC was also observed, which likely reflects the fact that Fy^a^ individuals tend to be Fy+. However, the hydroxyurea MTD was not associated with this genetic polymorphism, suggesting that treatment-related effects with mild myelosuppression are not influenced by this genetic pathway. Still unanswered is whether these polymorphisms are associated with an increased risk of neutropenia during hydroxyurea treatment and if they have any impact on clinical disease severity.

WES data were further examined to search for novel genetic variants associated with WBC and ANC, using a careful discovery and validation approach. Several genes with coding variants associated with either WBC or ANC were identified and validated (Table 2), but only a single *ZFHX4* variant was associated with both phenotypes (increased WBC and ANC). The *ZFHX4* gene encodes the zinc finger homeobox 4 protein that is involved in neuronal differentiation and possibly tumorigenesis.[37–39] To our knowledge this gene has not been previously implicated in hematopoiesis, so represents a potential new pathway for investigation in SCA.

The influence of candidate genes influencing renal phenotypes was also examined, since individuals of African descent have increased rates of non-diabetic kidney disease when compared to individuals of European ancestry.[22,40,41] Specific polymorphisms that are unique to persons of African ancestry have been identified; *APOL1* variants provide a survival advantage against trypanosomal disease, but the *APOL1* G1 and G2 variants have been associated with nephropathy, potentially due to altered lysosomal permeability causing podocyte damage.[42] Previous studies on adults with SCA found an association between *APOL1* polymorphisms and markers of renal disease including proteinuria and hemoglobinuria.[35,43] The current study is the first to investigate this association in an exclusively pediatric cohort, before the onset of nephropathy. The *APOL1* G1 polymorphism was associated with albuminuria; children heterozygous or homozygous for the G1 polymorphism had a significantly higher risk of albuminuria, while the G2 allele had a mild protective effect. Duffy antigen expression was also associated with a protective effect against the development of renal disease, which possibly reflects its known influence on leukocytes. The association between lower WBC and presence of albuminuria has been observed previously but not consistently.[44,45] Other potential candidate genes with reported influence on albuminuria in adults were not associated with microalbuminuria in these pediatric SCA cohorts.

In the WES analysis we chose to investigate GFR, and several genetic variants previously implicated in renal disease and organ damage were identified (Table 4). Two genes were associated with increased GFR (*AGGF1* and *CYP4B1*), while three genes were protective against elevated GFR (*CUBN*, *TOR2A*, and *PKD1L2)*. *AGGF1* is involved in TNF-α induced endothelial activation and has recently been linked with liver fibrosis.[46,47] *CYP4B1* is a cytochrome P450 monooxygenase that is expressed in the kidney and recently a long non-coding RNA (lncRNA) of *CYP4B1* was associated with nephropathy in diabetic patients.[48] Cubilin has previously been associated with albuminuria; two *CUBN* variants were tested as candidates but WES analysis identified another *CUBN* variant (rs111265129, I2189V) protective against renal damage. The *TOR2A* gene encodes salusin-β, which is a potent endogenous atherogenic factor produced and secreted by infiltrating macrophages. Locally produced salusin-β may also act on adjacent endothelial cells to accelerate inflammatory responses and on macrophages to induce foam cell formation.[49–51] A splicing variant was also identified in the *PKD1L2* gene, which is a member of the polycystin-1 family and highly homologous to the *PKD1* gene causing autosomal dominant polycystic kidney disease. *PKD1L2* is highly expressed in the kidney and is a suspected modifier of polycystic kidney disease,[52] so represents a plausible genetic modifier of sickle cell nephropathy.

A variant in *CD163* (rs61729510; S570N) is predicted to have a damaging effect on the encoded protein, and was associated with increased GFR. *CD163* encodes a receptor expressed exclusively in the monocyte-macrophage cell lineage, which mediates the uptake of hemoglobin-haptoglobin complexes.[53] Under conditions of high or chronic hemolysis, plasma haptoglobin is rapidly depleted and CD163 facilitates uptake of free hemoglobin and heme, and thus initiates a protective macrophage transcriptional response.[54] Functional studies are warranted since individuals with this variant may have reduced CD163 activity and be unable to remove free hemoglobin and heme adequately from circulation, which could increase inflammation and contribute to tissue injury.

Limitations of our analysis include the relatively young age of our patients, which presents a potential bias against the onset of phenotypes such as albuminuria, and the fact that GFR was measured in one cohort and calculated in the other, using both as surrogates for renal disease. Another important limitation is that our cohorts were not at steady-state at baseline, with a portion of children receiving chronic transfusion at the time of enrollment, however previous studies have demonstrated that elevated leukocyte counts persist despite chronic transfusion.[55] There is the potential for selection bias for children with more severe disease in all three studies; children in the SWiTCH cohort had suffered from stroke, children in TWiTCH had transitional TCDs, and children in HUSTLE cohort typically elected to enroll due to recurrent acute chest syndrome or vaso-occlusive crises. For this reason, as well as lack of ability to compare genotypes to children with less severe disease, correlations with clinical markers of disease severity were not performed. In addition, we did not analyze the potential confounding effects of renal medications such as ACE inhibitors or antihypertensive therapy, but these are not commonly used in children with SCA. Novel genes identified by WES maintained weak association in our validation cohort; however the lack of statistical adjustment for multiple comparisons increases the risk of false positives. Accordingly, it is important to consider these genetic variants for further investigation and validation. Despite these caveats, and similar to previous analysis of genetic factors affecting stroke risk in SCA,[56] most previously published polymorphisms for WBC and renal phenotypes could not be validated in these pediatric cohorts. This observation suggests that some genetic associations may be age-dependent, may provide only weak effects, or may not be correct. For this reason, concerted efforts must be made to independently replicate all published genetic findings, using adequately sized studies, before they can be considered valid modifiers. Especially for genetic variants originally identified in the general African American population, we observed few with significant effects in children with SCA. Only the *DARC* polymorphism was validated as a modifier of the WBC and ANC, and yet it had no discernible influence on the hydroxyurea MTD, and is unlikely to modulate clinical disease severity, so its utility as a clinically useful genetic predictor is questionable. However, our WES analysis, which included both a discovery and validation cohort, has identified several novel gene variants that warrant further investigation and validation in other cohorts.

## Supporting Information

S1 Table Summary of candidate genes analyzed. (DOCX)
