# Supplementary material for: Genetic Modifiers of White Blood Cell Count, Albuminuria and Glomerular Filtration Rate in Children with Sickle Cell Anemia
Source: PLoS One. 2016 Oct 6;11(10):e0164364. doi: 10.1371/journal.pone.0164364 (PMC5053442; doi:10.1371/journal.pone.0164364)
Supplement: S1 Table — (DOCX) [file pone.0164364.s001.docx]

| Cohort | Gene | SNP | Chromosome | Polymorphism | Method | Effect | Location |
| --- | --- | --- | --- | --- | --- | --- | --- |
| WBC | *DARC* | rs2814778 | 1q23 | T/C | Taq Man | Loss of function in *GATA* binding motif abolishes expression on erythroids | 5’UTR |
|  | *DARC* | rs12075 | 1q23 | A/G | Taq Man | Determines Duffy blood group antigen expression | Exon |
|  | *IFI16* | rs4657616 | 1q23 | A/G | Unidirectional Sanger Sequencing | Unknown effect, susceptibility biomarker | Intron |
|  | *PSMD3-CSF* | rs4065321 | 17q21.1 | C/T | Taq Man | Unknown effect, susceptibility biomarker | Intron |
|  | *CDK6* | rs445 | 7q21.2 | C/T | Taq Man | Unknown effect, susceptibility biomarker | 5’UTR |
|  | *CXCL2* | rs9131 | 4q13.3 | T/C | Taq Man | Unknown effect, susceptibility biomarker | Exon |
| Albuminuria | *APOL1*  G1 | rs73885319 | 22q12 | A/G | Taq Man | Alters structure of SRA Trypanosomal binding site | Exon |
|  | *APOL1* G2 | rs71785313 | 22q12 | In frame 6bp deletion | Bidirectional Sanger Sequencing | Alters structure of SRA Trypanosomal binding site | Exon |
|  | *eNOS* 4a | VNTR | 7q35 | 27bp repeat | PCR for VNTR | Unknown effect, susceptibility biomarker | Intron |
|  | *eNOS* | rs1799983 | 7q35 | G/T | Taq Man | Missense mutation, Unknown effect, susceptibility biomarker | Exon |
|  | *eNOS* | rs2070744 | 7q35 | T/C | Taq Man | Increase mRNA expression | Promoter |
|  | *CUBN* | rs7918972 | 10p12 | T/G | Taq Man | Missense mutation Unknown effect, susceptibility biomarker | Intron |
|  | *CUBN* | rs1801239 | 10p12 | T/C | Taq Man | Missense mutation, alters protein binding site | Exon |

**S1 Table. Summary of candidate genes analyzed**

Reference data obtained from single nucleotide polymorphism database (dbSNP). VNTR indicates variable number tandem repeat; bp, base pair; SRA serum-resistance associated protein; and UTR, untranslated region.
